# Self-efficacy scale for Brazilians with type 1 diabetes

**DOI:** 10.1590/S1516-31802007000200006

**Published:** 2007-03-04

**Authors:** Daniela Alves Gastal, Ricardo Tavares Pinheiro, Débora Potter Vazquez

**Keywords:** Diabetes mellitus, Scales, Self efficacy, Psychometrics, Patient, Diabetes mellitus, Escalas, Auto-eficácia, Psicometria, Paciente

## Abstract

**CONTEXT AND OBJECTIVE:**

Diabetes is a public health problem and good glycemic control is able to prevent or contain its complications. Self-efficacy is a key factor in successfully achieving behavior goals. The aim of this study was to analyze the psychometric properties of the insulin management diabetes self-efficacy scale (IMDSES) on type 1 diabetes patients from southern Brazil.

**DESIGN AND SETTING:**

Validation study in two cities in southern Brazil.

**METHODS:**

The psychometric properties of IMDSES were evaluated in a population of type 1 diabetes patients (n = 213), from September to December 2004, who were attended within the Brazilian public healthcare system. Principal component analysis was conducted to develop the subscales. Cronbach’s alpha was used as the reliability coefficient.

**RESULTS:**

The analysis of psychometric properties resulted in an IMDSES consisting of 20 items and three subscales: diet (alpha: 0.83), insulin (alpha: 0.92) and general management (alpha: 0.78) and accounted for 53% of the variance. Criteria validity was investigated through two parameters: glycohemoglobin, which showed significant association with self-efficacy on the insulin subscale (p = 0.04), and the variable "adherence", which was significantly associated with self-efficacy on two subscales (p < 0.05).

**CONCLUSIONS:**

This study shows that the IMDSES is valid and reliable, and can be used to measure results from diabetes educational programs and to measure self-efficacy relating to diabetes management, for possible interventions.

## RESUMO

**CONTEXTO E OBJETIVOS:**

Diabetes é um problema de saúde pública e sabe-se que o controle glicêmico adequado pode prevenir ou retardar as complicações e, sendo a auto-eficácia fator chave para atingir as metas comportamentais com sucesso, o objetivo deste estudo foi analisar as propriedades psicométricas da escala de auto-eficácia no manejo da insulina (IMDSES) em uma população de diabéticos tipo 1 do sul do Brasil.

**TIPO DE ESTUDO E LOCAL:**

Estudo de validação, realizado em uma população de diabéticos tipo 1 de duas cidades do sul do Brasil.

**METODOS:**

As propriedades psicométricas da IMDSES foram avaliadas em 213 diabéticos tipo 1, de setembro a dezembro de 2004, que eram atendidos pelo sistema único de saúde (SUS). A análise dos componentes principais para o desenvolvimento de subescalas e o coeficiente alfa de Cronbach foi utilizado na avaliação de confiabilidade da escala.

**RESULTADOS:**

Após analise das propriedades psicométricas, a escala ficou com 20 itens e três sub-escalas, dieta (alpha = 0,83), insulina (alpha = 0,92) e cuidados gerais (alpha = 0,78) que correspondiam a 53% da variância. Validade de critério foi investigada através de dois parâmetros: glicohemoglobina, através da qual foi possível verificar associação significativa com auto-eficácia na subescala insulina (p = 0,04) e com a variável "adesão", que foi significativamente associada com auto-eficácia em duas sub-escalas (p < 0,05).

**CONCLUSÃO:**

Este estudo verificou que IMDSES é válida e confiável e pode ser utilizada para avaliar resultados de programas educacionais em diabetes e auto-eficácia no manejo do diabetes para possíveis intervenções.

## INTRODUCTION

Diabetes mellitus is a public health problem both in developed and in develo-ping countries. There are about 5 million diabetics in Brazil, although half of them are unaware of their condition.^1^ It has been estimated that, by 2025, this disease will affect 11.6 million Brazilians and that Brazil will be among the 10 countries with the highest prevalence of this disease. Diabetes is among the 10 major mortality causes in the country.^2^

It is well documented that individuals with type 1 diabetes face a complex treatment regimen after their disease has been diagnosed, encompassing a series of daily decisions that demand active behavioral involvement by the patient. One study has indicated that patients consider the daily self-care regimen to be more difficult than the diagnosis.^3^ Adherence to treatment is low according to data in the literature,^4^ and inadequate control of the disease may lead to acute and chronic complications in several organs, such as the kidneys, eyes and heart, and in the circulation.^5,6^ Therefore, it is important to have good glycemic control, and self-efficacy is the key factor in successfully achieving behavioral goals.

According to Bandura, self-efficacy reflects the individual’s belief in his/her ability to successfully perform specific activities. Belief influences goals, motivation and perseverance relating to the quality of analytical thought and causal attribution of success and failure.^7^ The sense of self-efficacy is based on four sources: the individual’s own significant experiences, resulting from interpreting proposed performance; vicarious experiences that alter opinions about efficacy through transmission of competencies and comparison with other patients’ accomplishments; verbal persuasion that the individual possesses certain abilities, and similar types of social influence; and physiological and affective states, from which people partially judge their abilities, resistance and vulnerability to dysfunction.^8^ Belief in personal ability to undertake certain behavior influences choices, situations that will be attempted or avoided and perseverance in performing the task.^9^

The value of self-efficacy in predic-ting self-care behavior relating to diabetes has been verified in the literature, through studies in which self-efficacy was associated with self-reporting of adherence to treatment among adolescents^10-15^ and adults.^16,17^ It has been related to good glycemic control^18^ and to perceived improvement of general health and social functioning.^19^

Self-efficacy has been successfully used in educational programs for diabetes patients.^20-23^ The lack of instruments adapted and validated for the Brazilian population has been a barrier for conducting studies relating to this population. We found in the literature many instruments that evaluate self-efficacy in diabetes cases: the Diabetes Empowerment Scale (DES) and the Diabetes Self-Efficacy Scale (DSES), which evaluate self-efficacy in type 1 and 2 diabetics;^24,25^ the Self-Efficacy in Adolescent Girls and Boys With Insulin-Dependent Diabetes Mellitus scale, which is specific for type 1 diabetics;^16^ and the Insulin Management Diabetes Self-Efficacy Scale (IMDSES).

The instrument chosen for adaptation was the IMDSES, which is from the United States and was developed by Hurley and Harvey to evaluate self-efficacy among diabetics.^26^

## OBJECTIVE

This study was carried out in order to translate, adapt and evaluate the psychometric properties of the IMDSES, in a sample of type 1 diabetics from southern Brazil.

## METHODS

### Sample

The sample was made up of 213 individuals. The sample size was calculated using an average of 7 individuals per scale item with a margin of 10%.^27^ The participants were individuals with a diagnosis of type 1 diabetes who were registered within the Brazilian national health system (Sistema Único de Saúde, SUS) in two cities in southern Brazil. The study was conducted between September and December 2004.

The patients were selected at random according to their respective registrations in public service pharmacies that provided insulin. The inclusion criteria were: age of 14 years or older (data in the literature indicates that from this age on the individual realizes his/her need for self-help^16^); type 1 diabetes (defined as beginning before reaching 40 years of age and treated with insulin from the time of diagnosis); diabetes diagnosed more than six months before the study period; and self-care of diabetes, meaning that the patients were looking after themselves without the aid of third parties. Pregnant women were excluded.

### Original instrument

The IMDSES instrument evaluates self-efficacy relating to self-care of diabetes, among subjects who need to take insulin. It was devised in the United States and contained 28 items, with questions for which the responses were on a Likert-like scale of six points, from 1 (strongly agree) to 6 (strongly disagree).^28^ Seven types of behavior were evaluated: general management, diet, exercise, care with feet, glycemia monitoring, insulin administration and detection, prevention or treatment of hypoglycemia/hyperglycemia. It was originally validated among 142 adults who were insulin-requiring diabetics. The psychometric properties of the original version were adequate. Cronbach’s alpha for the whole scale was α = 0.82, and for the three domains (described below) it was: general management, α = 0.67; diet, α = 0.78; and insulin, α = 0.77. The test-retest stability was acceptable (r = 0.58, p < 0.002) and was followed up two weeks later.

An item-by-item self-reported assesment instrument called the insulin management diabetes self-care scale (IMDSCS) was developed and applied to all participants, before they responded to the IMDSES and underwent the glycohemoglobin test.^26^

### Procedures for adaptation

After approval of the study by the Research Ethics Committee of Hospital Santa Casa de Misericórdia de Pelotas, in Pelotas, Rio Grande do Sul, the work of adapting the instrument started from a theoretical analysis of the items.^27^

The instrument was translated from English to Portuguese by two bilingual experts, and then a back translation was made by other two translators who had not seen the original scale, in order to verify the equivalence of terms between the two versions.

For the theoretical analysis of the items, 20 doctors from Rio Grande do Sul who were specialists in diabetes evaluated the adequacy of these items and their pertinence to the respective beha-viours. Any item with an 80% agreement between these judges was considered satisfactory.^26^

Semantic analysis was done among 10 insulin-requiring patients, to analyze their understanding of the questions and instructions of the scale, and then a pilot study was conducted with another 10 patients. In this study, we found that there was some difficulty in understanding some items about insulin management, but not with regard to type 1 diabetics, and therefore we decided to adapt the scale for use only among type 1 diabetics.

Some changes were made to the instrument to improve understanding. All the items were set in an affirmative mode, whereas the original instrument had positive and negative items. We changed the Likert scale to use only four points: 1- strongly agree; 2- agree; 3- disagree; and 4- strongly disagree. We also introduced the possibility of marking a fifth alternative, named "not applicable", which was computed as *missing information*. Items 6 and 10 were changed by adding examples. The final instrument is presented in Annex 1.

For interpreting the scale, reverse scoring was used. This corresponded to the mean scores computed for each item of the respective subscale. As in the original scale, no cutoff point was recommended in this scale, because self-efficacy is a dynamic concept. If we had had to recommend a cutoff point it would have been located in the upper third of the total score for the scale, because within this range there was greater correlation with high levels of self-efficacy in the statistical analysis.

To the initial instrument was added a questionnaire on the sociodemographic characteristics of the study population, which included the following questions: name, address, telephone number, skin complexion, birth date, sex, schooling in completed years of study, social class as evaluated by the criteria of the Brazilian Association for Population Studies (Associação Brasileira de Estudos Populacionais, Abep), date of diabetes diagnosis, date of starting insulin use, types of insulin used, amounts of insulin and number of bottles used in a month, and whether the individual carried out his/her self-help without the aid of third parties.

After agreeing to take part in the study, the patients signed a consent form and, in the case of patients under 18 years of age, their parents gave their consent in writing. The data were collected in the laboratory recommended by the researcher or in the homes of interviewees who lived out of town or who had difficulty in reaching the indicated location. The instrument was administered by previously trained psychology students from the Catholic University of Pelotas (Universidade Católica de Pelotas, UCPEL) in the form of a dialogue interview. Blood was collected to carry out the glycohemoglobin test.

Two months later, the retest was performed on 54 patients who were selected randomly from among the initial sample.

### Data analysis

The Statistical Package for the Social Sciences (SPSS) for Windows software, version 8.0,^29^ was used for processing the analysis.

To study the construct validity of the instrument, i.e. whether the test does in fact measure what it proposes to, multivariate analysis was carried out using principal components analysis.^30,31^

Univariate analysis (frequencies and means) was conducted to observe the distribution and to establish cutoff points when necessary, both for sociodemographic variables and for subscales of the instrument.

To study criterion validity, two parameters were utilized: glycohemoglobin (GHb) and "adherence". Unlike in the original IMDSES scale, the IMCSCS scale was not used because this is not considered to be valid in Brazil. GHb was measured using the high-performance liquid chromatography (HAPLY) method through ion exchange (reference: < 7%) in a single laboratory and was treated as an ordinal variable. The variable "adherence", which is behavioral, was measured by calculating the relationship between number of units used and number of insulin bottles consumed during the period. The latter was given by an external criterion (number of bottles supplied by pharmacies to diabetics during that period). Therefore, a categorical variable was established (yes or no). Analogies for using this criterion was made with studies that have correlated adherence with the accurate method of counting pills.^32-35^

It was established that, for statistical analysis, "self-efficacy" would be converted into a variable divided into terciles. For the ordinal variable GHb we used analysis of variance (ANOVA), and for the "adherence" variable, the chi-squared test (bivariate) and logistic regression (multivariate) to evaluate possible confounding factors in the results.

The reliability of the instrument was determined by internal consistency analysis, using Cronbach’s alpha. To evaluate temporal stability we used test-retest and Pearson’s correlation.

## RESULTS

### General characteristics of the sample

221 subjects had been selected to take part in the study, but eight dropped out: one because he was dependent on third-party help and seven because they refused to take part. Thus, the resultant final sample was 213, of which 47.9% were men and 52.1% were women. Their mean age was 33.9 ± 14.97 years and most of them were fair-skinned (90.1%). The predominant social class was C (44.1%), and the mean schooling level in completed years of study was 8.9 ± 0.27 years. The length of time since diagnosis was 12.1 years. The marital status of 49% was single. The mean GHb level was 10.28 ± 2.3%.

The retest sample comprised 54 patients. This sample presented some differences in characteristics, in relation to the test sample, with majorities of men (58.2%) and singles (61.8%). The mean age was lower: 30.9 ± 15.2 years. The predominant social classes were B and C (73%) and 45% had studied for up to eight years. The mean length of time since diagnosis was 11.4 years and the mean GHb level 9.7 ± 2.6 %. However, the differences between the test and retest samples were not significant.

### Psychometric characteristics of the instrument

In the initial analysis with 28 items, it was found that eight of them presented factorial loads of less than 0.30, which led to their removal.

In the study of construct validity with 20 items, factorability was shown by KMO coefficient of 0.86, Bartlett’s sphericity test (p < 0.001), significant inter-item correlation coefficients (p < 0.001) and communalities that oscillated from 0.55 to 0.86.

**Figure 1:**
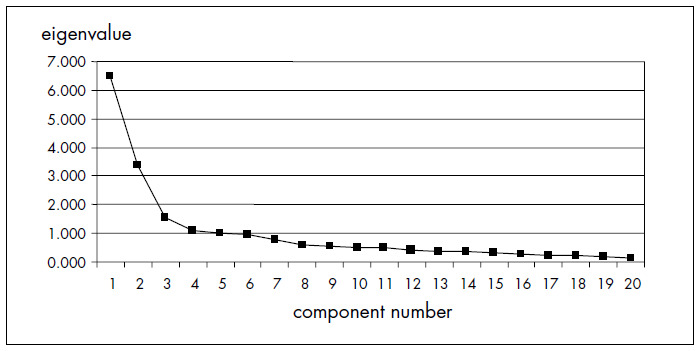
Scree plot: eigenvalues versus component numbers.

Five components presented eigenvalues greater than 1.0 in the initial principal components solution. The scree plot showed three distinct visual components that accounted for 53.6% of the variance (Figure 1). After conside-ring the interpretability of the components and analyzing possible solutions, it was decided to extract three subscales (diet, insulin and general management). Their factorial loads ranged from 0.49 to 0.90, and it had been previously decided that only items with factorial loads greater than 0.30 would be considered^27^ (Table 1).

**Table 1:** Psychometric parameters of the adapted instrument (n = 213)

	Components		
	Diet subscale	Insulin subscale	General management subscale
	1	2	3
1	0.52		
5	0.53		
6	0.73		
7	0.69		
8	0.72		
9	0.68		
10	0.55		
11	0.67		
14		0.88	
15		0.90	
16		0.79	
17		0.88	
18		0.76	
02			0.78
03			0.80
04			0.50
12			0.52
13			0.61
19			0.49
20			0.59
Eigenvalue	5.5	3.4	1.6
Total variance (%)	27.9	17.2	8.4
Number of items	8	5	7
Internal consistency (α)	0.83	0.92	0.78

**Figure 2:**
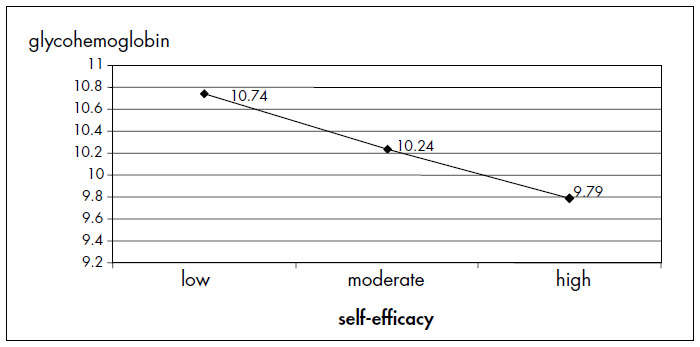
Analysis of variance for glycohemoglobin (GHb) and self-efficacy measured on the insulin subscale. Glycohemoglobin corresponding to low self-efficacy is 10.74 ± 2.66%; moderate 10.24 ± 2.66%; and high 9.79% ± 2.23%.

In the criterion validity analysis for the GHb level, ANOVA testing on the insulin subscale was significant (p = 0.040), showing a linear tendency (p = 0.01) between groups: the higher the self-efficacy level was, the lower the GHb level was (Figure 2). Regarding "adherence", it was seen that the higher it was, the higher the levels of self-efficacy were on the two subscales of diet and insulin. The chi-squared test showed a significant association on the subscales of diet (p = 0.04) and insulin (p = 0.03), but not on the general management subscale (p = 0.24). In the logistic regression analysis, after adjustment for age, sex, social class and schooling, it was seen that subjects with low self-efficacy had approximately twice the chance of showing "non-adherence" on the subscales of diet and insulin, in comparison with the 33% who showed the best self-efficacy on these subscales.

The reliability of the instrument was determined by internal consistency analysis using Cronbach’s alpha (α), which verified whether the subscales identified were consistent and homogeneous. The coefficients were as follows: diet (α = 0.83), insulin (α = 0.92) and general management (α = 0.78).

The test-retest technique verified the scale stability over a two-month period. This was evaluated using Pearson’s correlation, giving the following subscale results: diet (r = 0.33; p = 0.01), insulin (r = 0.13; p = 0.30) and general management (r = 0.61; p = 0.00).

## DISCUSSION

The results from this investigation rela-ting to psychometric properties are promising. The content validity, as verified by theoretical analysis of the items and semantic analysis of the scale, was adequate. In the semantic analysis, we tried to evaluate the items with subjects from extremes of social classes, so that the language would be adequate for all levels, as recommended in the literature.^27^ In the pilot study, it was decided to make changes in three items and to reduce the number of scores on the Likert scale, in order to improve understanding. Difficulty in understanding Likert-like scales with many score options have been reported in the literature among populations of low socioeconomic level.^28^

Regarding construct validity, three subscales were maintained, as in the original instrument, but the number of items was reduced to 20 and the instrument obtained more reliable measurements. The wording of item number one was modified, after being identified in the diet subscale. In the original instrument, it had belonged to the general management subscale (Annex 1).

Among the parameters used for analyzing the external criterion validity, GHb is an important test that reflects the mean glycemic control over the preceding three months. Its mean in our sample was above the recommendations for the method (10.28 ± 2.33) and the mean from the original scale (10.99 ± 2.36), thus showing ina-dequate metabolic control in both populations.

If the GHb level is adequate, it may prevent or delay diabetes complications.^36,37^ It was found that there was a significant association between the self-efficacy level on the insulin subscale and GHb, which was confirmed by ANOVA. There was a linear trend in which the higher the self-efficacy level was, the lower the GHb level was. In the original instrument, there were significant associations with GHb on all subscales, which was not observed in the present study.

Studies in the literature have correlated self-efficacy with glycemic control using GHb^14,15^ but, according to Glasgow et al. (1999),^36^ it is inappropriate to use GHb as an indicator for a patient’s behavior. High GHb may be attributable to behavioral problems or other factors (inadequate prescription or diseases, among others) that must be analyzed. Adherence is an important contributor towards good control, but it is not the same as control and should not be interpreted on the basis of a single laboratory test.^36^ After searching for a behavioral characteristic that would reflect diabetes management, the variable "adherence" was defined. It was observed that there was a significant association between "adherence" and self-efficacy in the correlation analysis, on the subscales of diet (p < 0.02) and insulin (p < 0.02). This was also found in the logistic regression analysis, thus indicating that the scale has criteria validity, i.e. the subscales of diet and insulin predict "adherence" or diabetes management. Hence, in the multivariate analysis, after adjustment for age, sex, schooling and social class, and using a cutoff point of 33% for subjects with better self-efficacy levels, there was a significant association between self-efficacy and "adherence" (adequate behavior regarding the use of insulin and diet).

In the reliability study, Cronbach’s alpha for the subscales ranged from 0.92 for insulin factor to 0.78 for general management. Alpha values can range from 0 to 1, and 0.65 is the lowest acceptable value for the scale to be considered reliable.^38^ But considering 0.80 as the expected index for a measurement with less error, the value for the general management subscale was acceptable.^27^

The evaluation of temporal stability using test-retest did not show any significant association with the insulin subscale. It is likely that the two-month period was too long and interfered with the results, considering that self-efficacy is a dynamic concept and can change with time. In the original instrument, the second test was performed after two weeks because of this possibility.

There is evidence that behavioral patterns relating to diabetes care are relatively independent. Characterization for the specific behavioral area that presents impairment allows more effective interventions.^37^ It is known that self-efficacy is specific for each behavioral pattern,^7^ thus explaining the findings from this study.

Our study presented certain limitations such as the way in which the instrument was applied: it was done by interview and not self-application as the original, and this may have affected the replies.^27^ Considering that the original instrument was designed for insulin-requiring diabetics, whereas for our population it was decided to adapt it only for type 1 diabetics, no generalization for all diabetics is possible.

## CONCLUSION

It was concluded that, despite the modifications made to the instrument, in relation to the original questionnaire, there was no loss in internal consistency in its adaptation. It presents adequate psychometric parameters and can be used to evaluate self-efficacy in the management of type 1 diabetes.

It is suggested that further studies in different regions of the country should be conducted to take the cultural diversity into account, using different parameters to verify the criterion validity, as well as several samples of diabetics with investigation of temporal stability over a shorter time interval.

## Appendix

**Annex 1:** Final instrument

Questions	Strongly Agree	Agree	Disagree	Strongly Disagree	Not applicable
1. I can follow the diet most of the time in my routine.	1	2	3	4	NA
2. I believe in my ability to deal with diabetes.	1	2	3	4	NA
3. I feel confident in using my knowledge about diabetes in my daily treatment.	1	2	3	4	NA
4. I believe I can follow diabetes routines daily.	1	2	3	4	NA
5. I can have the meals at the same time every day.	1	2	3	4	NA
6. I can maintain my diet when eating outside of my home in known places (e.g. a friend’s house).	1	2	3	4	NA
7. I can maintain my diet when eating outside of my home in unknown places.	1	2	3	4	NA
8. I am sure I will be able to maintain my diet when the people around me don’t know I am diabetic.	1	2	3	4	NA
9. I am sure I can maintain my diet every day.	1	2	3	4	NA
10. I can correctly replace one type of food with another in the same group (e.g. replacing rice with potato).	1	2	3	4	NA
11. I can maintain my diet when I attend parties.	1	2	3	4	NA
12. I can apply insulin using the correct technique.	1	2	3	4	NA
13. I have the capability of applying insulin when I am not at home.	1	2	3	4	NA
14. I can adjust my insulin amount on the basis of the results from sugar tests in blood or urine, when necessary.	1	2	3	4	NA
15. I am sure I can adjust my insulin amount when there are changes in my daily routine.	1	2	3	4	NA
16. I know how to adjust my insulin amount in order to avoid hypoglycemia when I am exercising.	1	2	3	4	NA
17. I know what type of adjustment in the insulin amount I have to make when my blood sugar is higher than it should be.	1	2	3	4	NA
18. I can adjust my insulin amount when I have a cold or flu.	1	2	3	4	NA
19. I am sure diabetes treatment does not interfere with my daily routine.	1	2	3	4	NA
20. I think I am capable of following the planned treatment for diabetes, even when there are changes in my daily routine.	1	2	3	4	NA

## References

[1] Malerbi DA, Franco LJ (1992). Multicenter study of the prevalence of diabetes mellitus and impaired glucose tolerance in the urban Brazilian population aged 30-69 yr. The Brazilian Cooperative Group on the Study of Diabetes Prevalence. Diabetes Care.

[2] Chacra AR, Moisés RCMS, Coronho V, Petroniau A, Santana EM, Pimenta LG (2001). Diabetes melito: classificação e diagnóstico. Tratado de endocrinologia e cirurgia endócrina.

[3] Anderson RM (1985). Is the problem of noncompliance all in our heads?. Diabetes Educ.

[4] Ary DV, Toobert D, Wilson W, Glasgow RE (1986). Patient perspective on factors contributing to nonadherence to diabetes regimen. Diabetes Care.

[5] The effect of intensive treatment of diabetes on the development and progression of long-term complications in insulin-dependent diabetes mellitus (1993). The Diabetes Control and Complications Trial Research Group. N Engl J Med.

[6] Intensive blood-glucose control with sulphonylureas or insulin compared with conventional treatment and risk of complications in patients with type 2 diabetes (UKPDS 33) (1998). UK Prospective Diabetes Study (UKPDS) Group. Lancet.

[7] Bandura A (1997). Self-efficacy: The exercise of control.

[8] Maibach E, Murphy DA (1995). Self-efficacy in health promoting research and practice: conceptualization and measurement. Health Educ Res.

[9] Bandura A (1977). Self-efficacy: toward a unifying theory of behavioral change. Psychol Rev.

[10] McCaul KD, Glasgow RE, Schafer LC (1987). Diabetes regimen behaviors. Predicting adherence. Med Care.

[11] Glasgow RE, Toobert DJ, Riddle M, Donnelly J, Mitchell DL, Calder D (1989). Diabetes-specific social learning variables and self-care behaviors among persons with type II diabetes. Health Psychol.

[12] Hurley AC, Shea CA (1992). Self-efficacy: strategy for enhancing diabetes self-care. Diabetes Educ.

[13] Padgett DK (1991). Correlates of self-efficacy beliefs among patients with non-insulin dependent diabetes mellitus in Zagreb, Yugoslavia. Patient Educ Couns.

[14] Aalto AM, Uutela A (1997). Glycemic control, self-care behaviors, and psychosocial factors among insulin treated diabetics: a test of an extended health belief model. Int J Behav Med.

[15] Kavanagh DJ, Gooley S, Wilson PH (1993). Prediction of adherence and control in diabetes. J Behav Med.

[16] Grossman HY, Brink S, Hauser ST (1987). Self-efficacy in adolescent girls and boys with insulin-dependent diabetes mellitus. Diabetes Care.

[17] Littlefield CH, Craven JL, Rodin GM, Daneman D, Murray MA, Rydall AC (1992). Relationship of self-efficacy and binging to adherence to diabetes regimen among adolescents. Diabetes Care.

[18] Griva K, Myers LB, Newman S (2000). Illness perceptions and self efficacy beliefs in adolescents and young adults with insulin dependent diabetes mellitus. Psychology and Health.

[19] Aalto AM, Uutela A, Aro AR (1997). Health related quality of life among insulin-dependent diabetics: disease-related and psychosocial correlates. Patient Educ Couns.

[20] Glasgow RE, Osteen VL (1992). Evaluating diabetes education. Are we measuring the most important outcomes?. Diabetes Care.

[21] Johnson JA (1996). Self-efficacy theory as a framework for community pharmacy-based diabetes education programs. Diabetes Educ.

[22] Engel SS, Crandall J, Basch CE, Zybert P, Wylie-Rosett J (1997). Computer-assisted diabetes nutrition education increases knowledge and self-efficacy of medical students. Diabetes Educ.

[23] Corbett CF (1999). Research-based practice implications for patients with diabetes. Part II: Diabetes self-efficacy. Home Healthc Nurse.

[24] Anderson RM, Funnell MM, Butler PM, Arnold MS, Fitzgerald JT, Feste CC (1995). Patient empowerment. Result of a randomized controlled trial. Diabetes Care.

[25] Crabtree MK (1986). Self-efficacy and social support as predictors of diabetic self-care. [Dissertation].

[26] Hurley AC, Harvey RM, Strickland O, Dilorio C (1990). The Insulin Management Diabetes Self-efficacy Scale. Measurement of nursing outcomes.

[27] Pasquali L, Pasquali L (1990). Testes referentes à construto: teoria e modelo de construção. Instrumentos psicológicos: manual prático de elaboração.

[28] Statistical Package for the Social Sciences [computer program] (1998). SPSS base 8.0 for Windows.

[29] Floyd FJ, Widaman KF (1995). Factor analysis in development and refinement of clinical Assessment Instruments. Psychological Assessment.

[30] Zwick WR, Velicer WF (1986). Comparison of five rules for determining the number of components to retain. Psychol Bull.

[31] Balakrishnan S, Kumar A, Rao BR, Patro TP (1986). Implementation of tests for monitoring drug compliance of leprosy out-patients under multi-drug therapy. Indian J Lepr.

[32] Botelho RJ, Dudrak R (1992). Home assessment of adherence to long-term medication in the elderly. J Fam Pract.

[33] Farmer KC (1999). Methods for measuring and monitoring medication regimen adherence in clinical trials and clinical practice. Clin Ther.

[34] Thompson C, Peveler RC, Stephenson D, McKendrick J (2000). Compliance with antidepressant medication in the treatment of major depressive disorder in primary care: a randomized comparison of fluoxetine and tricyclic antidepressant. Am J Psychiatry.

[35] Bernal H, Wooley S, Schensul JJ (1997). The challenge of using Likert-type scales with low-literate ethnic populations. Nurs Res.

[36] Glasgow RE, Fisher EB, Anderson BJ (1999). Behavioral science in diabetes. Contributions and opportunities. Diabetes Care.

[37] Van Der Ven NC, Weinger K, Yi J (2003). The confidence in diabetes self-care scale: psychometric properties of a new measure of diabetes-specific self-efficacy in Dutch and US patients with type 1 diabetes. Diabetes Care.

[38] DeVellis RF (1991). Scale development: theory and applications. (Applied Social Research Methods).

